# The Enigmatic Conserved Q134-F135-N137 Triad in SARS-CoV-2 Spike Protein: A Conformational Transducer?

**DOI:** 10.3390/biom16010111

**Published:** 2026-01-08

**Authors:** Marine Lefebvre, Henri Chahinian, Nouara Yahi, Jacques Fantini

**Affiliations:** 1 IHU Méditerranée Infection, 19-21 Boulevard Jean Moulin, 13005 Marseille, France; marine.lefebvre@etu.univ-amu.fr; 2 Microbes Evolution Phylogeny and Infections (MEPHI), Aix-Marseille University, 27 Boulevard Jean Moulin, 13005 Marseille, France; 3 Department of Biology, Faculty of Medicine, INSERM UA16, Aix-Marseille University, 13015 Marseille, Francenouara.yahi@univ-amu.fr (N.Y.)

**Keywords:** SARS-CoV-2, lipid rafts, gangliosides, conformational wave, allosteric mechanism, evolution, infection, quantum mechanisms

## Abstract

Lipid raft-associated gangliosides facilitate the early stages of SARS-CoV-2 entry by triggering the exposure of the receptor-binding domain (RBD) within the trimeric spike protein, which is initially sequestered. A broad range of in silico, cryoelectron microscopy and physicochemical approaches indicate that the RBD becomes accessible after a ganglioside-induced conformational rearrangement originating in the N-terminal domain (NTD) of one protomer and propagating to the neighboring RBD. We previously identified a triad of amino acids, Q134-F135-N137, as a strictly conserved element on the NTD. In the present review, we integrate a series of structural and experimental data revealing that this triad may act as a conformational transducer connected to a chain of residues that are capable of transmitting an internal conformational wave within the NTD. This wave is generated at the triad level after physical interactions with lipid raft gangliosides of the host cell membrane. It propagates inside the NTD and collides with the RBD of a neighboring protomer, triggering its unmasking. We also identify a chain of aromatic residues that are capable of controlling electron transfer through the NTD, leading us to hypothesize the existence of a dual conformational/quantum wave. In conclusion, the complete conservation of the Q134-F135-N137 triad despite six years of extensive NTD remodeling underscores its critical role in the viral life cycle. This triad represents a potential Achilles’ heel within the hyper-variable NTD, offering a stable target for therapeutic or vaccinal interventions to disrupt the conformational wave and prevent infection. These possibilities are discussed.

## 1. Role of Lipid Rafts in SARS-CoV-2 Infection

Lipid rafts are specialized, cholesterol- and glycosphingolipid-enriched microdomains in the plasma membrane that serve as critical organizers of signaling, trafficking, and membrane dynamics [1,2,3,4,5,6]. Their unique composition [7,8] promotes the clustering of protein, gangliosides—sialic acid-containing glycosphingolipids—and cholesterol into condensed microdomains with a strong electronegative surface potential [9,10,11,12,13]. This configuration renders lipid rafts capable of initiating viral attachment through electrostatic interactions that attract positively charged amino acid residues present on the viral surface [14,15,16,17,18,19]. Such interactions represent the earliest stage of virion binding, concentrating viruses on the cell surface and facilitating subsequent high-affinity receptor engagement and internalization [20].

**Figure 1:**
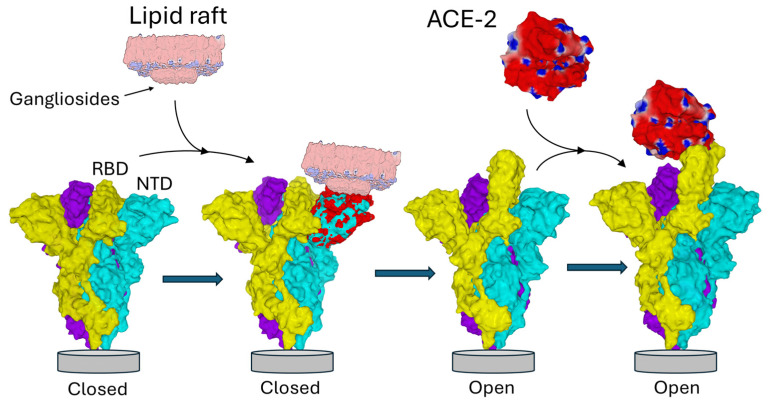
NTD-ganglioside-induced conformational change in the Spike trimer. The three subunits of the Spike trimer are colored cyan, yellow, and purple. Each NTD is located at the periphery of the trimer. Following the binding of one NTD to a lipid raft enriched in gangliosides, a conformational change propagates throughout the involved subunit (the one in cyan in this example) until it reaches the neighboring subunit (colored yellow). Areas of the NTD whose conformation has been modified after interaction with the raft are indicated in red. Note that the conformational rearrangements affect distant domains of the NTD, including the contact zone with the neighboring subunit. This triggers the unmasking of the RBD of the yellow subunit, now able to bind the ACE-2 receptor. Overall, this process is functionally reminiscent of interfacial activation [60]. Trimeric structures in the closed pre-fusion conformation were constructed with Swiss-PdbViewer [61] by homology with a reference model (pdb: 6VSB) [62]. ACE2 and the spike trimer in the open conformation were from pdb 7DK3 [37]. Protein structures were generated with Molegro Molecular Viewer 2.5 (MMV, http://molexus.io/molegro-molecular-viewer/ accessed on 3 March 2025).

Enveloped viruses, including coronaviruses, exploit lipid rafts as portals for cell entry [21,22,23,24,25,26,27]. Coronaviruses’ spike (S) glycoprotein mediates this process by recognizing not only protein receptors but also ganglioside-rich domains within lipid rafts [28,29,30,31,32,33,34]. SARS-CoV-2 is a perfect example of molecular duality. Organized in trimers in the virus envelope, this protein has two functional domains of interaction with the membrane of target cells: the N-terminal domain (NTD) and the receptor binding domain (RBD) [35,36]. The NTD is immediately functional while the RBD is initially masked in the trimer [37]. The interaction of the NTD with the plasma membrane of the target cell triggers an irreversible conformational change that unmasks the RBD, allowing the virus to interact with its ACE-2 receptor [20], in a typical interfacial activation process (Figure 1). Neutralizing antibodies directed against the NTD and against the RBD effectively block infection [38,39], even if the neutralization window is much wider for anti-NTD antibodies [40,41] whose main epitope is permanently accessible, which is not the case for the RBD [42,43].

Structural and molecular modeling studies have identified a large and flat ganglioside-binding domain (GBD) in the NTD [44,45], which forms energetically favorable complexes with gangliosides and is critical for viral attachment efficiency [14]. Blocking this interaction disrupts spike localization to lipid rafts and inhibits viral entry [46,47], underscoring lipid rafts’ key role in SARS-CoV-2 infectivity [48,49,50] and pathogenesis [51,52]. The interaction of the NTD with gangliosides (or with sialic acid-containing glycans) have been demonstrated in silico [33,53,54,55], but also experimentally with various approaches including surface plasmon resonance [28] and virus attachment assays [47]. NTD mutants or NTD-directed neutralizing antibodies such as 4A8 abolish this allosteric shift, linking ganglioside engagement to enhanced ACE2 exposure [47]. Moreover, Langmuir-based physicochemical approaches [56,57,58] showed that trimeric Spikes in the closed conformation from Wuhan, Alpha, Delta and Omicron variants interacted with ganglioside monolayers at the air–water interface [59]. Since only NTDs are accessible in these trimeric spikes, we can reasonably conclude that these monolayer experiments also show a raft–NTD interaction.

The spike exists in a metastable prefusion state where its three RBDs oscillate between “down” (closed) conformations—occluding receptor access—and “up” (open) conformations that expose the RBD for receptor binding [63]. Transition to the open state involves disruption of inter-subunit salt bridges, such as that between residue D614 and K854 [64], enabling one or more RBDs to adopt an erect position competent for ACE2 interaction. This conformational shift is further modulated by viral mutations (e.g., D614G) [65,66,67,68] and secondary interactions with host proteases that prime spike for membrane fusion [69,70].

Overall, this coordinated sequence—from ganglioside-mediated initial attachment within lipid rafts to the conformational activation of the spike trimer—illustrates the pivotal role of lipid raft microdomains in orchestrating the first critical phases of coronavirus infection [22,48,50,71,72,73,74,75,76]. However, this phenomenon has not been described in detail at the molecular level. In this review, we ask two essential questions: (i) is this mechanism still active in successive variants of SARS-CoV-2, including the variants currently circulating at the end of 2025; (ii) how can interaction with the gangliosides of a raft generate a conformational wave that propagates through the NTD to reach the neighboring protomer and trigger the RBD unmasking process? In this perspective, we will first characterize the GBD of the initial strain of SARS-CoV-2 that appeared in 2019. Secondly, we will study how NTD has evolved over the last six years. Then we will focus our study on a triad of conserved amino acids (Q134/F135/N137) located at the NTD interface in contact with the gangliosides of the raft on the host cell surface. We will see that this triad remains unchanged despite the accumulation of many mutations on the NTD surface, which strongly impacts the electrostatic potential of the ganglioside-binding domain (GBD). Finally, we will decipher the mechanisms by which raft gangliosides trigger a conformational wave leading to RBD unmasking. This conformational wave is superimposed on a quantum wave that can ensure electron transfer across the aromatic amino acids of the NTD. We believe that this overview of the molecular mechanisms linking rafts to conformational rearrangements of the SARS-CoV-2 trimeric spike protein will impact our understanding of the earliest stages of infection by this virus. These data could suggest new therapeutic and vaccine approaches centered on the Q134/F135/N137 triad.

## 2. Structural and Functional Characteristics of the GBD in the Initial Strain of SARS-CoV-2

**Figure 2:**
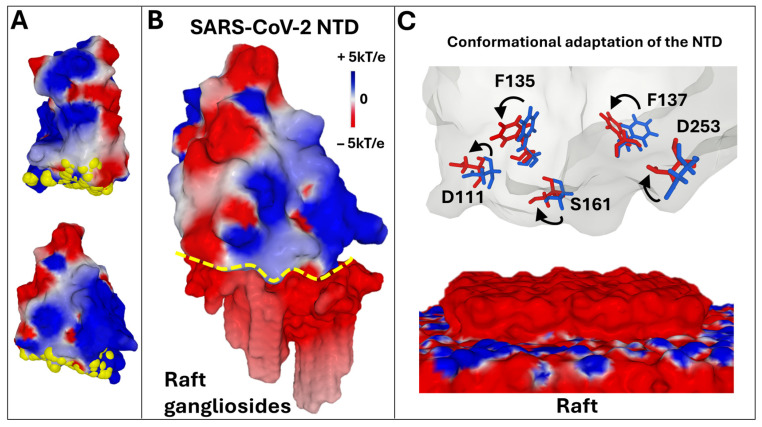
Main characteristics of the GBD in the initial strain of SARS-CoV-2. (**A**). The amino acids of the GBD (represented as yellow atomic spheres) form a large flat surface at the tip of the NTD (represented in two distinct orientations as electrostatic potential: blue, positive; red, negative; white, neutral). (**B**). NTD–raft complex. The two partners are represented in surface electrostatic potential. The interaction zone is marked with a dotted yellow boundary line. (**C**). The raft–NTD interaction mechanism is adaptive. Conformational changes involving GBD amino acids, such as those indicated by arrows, are highlighted on the NTD surfaces. These structural changes are induced by the movements of raft gangliosides, which can then be described as a molecular quicksand [14]. The electrostatic surface potential of the spike trimers was visualized with Molegro Molecular Viewer (MMV) (http://molexus.io/molegro-molecular-viewer) as described previously [82]. The electrostatic potential measured and illustrated by MMV is the sum of the Coulomb potential for each atom of the considered molecule, with a distance-dependent dielectric constant. It is represented in three colors: blue, electropositive, red, electronegative, white, neutral.

In the classification of the different GBD categories, that of the Spike protein of SARS-CoV-2 has been defined as a class 3 GBD (GBD-3) [44]. This type of GBD characterizes a large flat surface of interaction with several gangliosides. In the case of the NTD of the initial strain of SARS-CoV-2 that appeared in 2019, the GBD occupies an area estimated at ≈10 nm^2^ which represents roughly 70% of the solvent accessible surface of the NTD tip (Figure 2A). The electrostatic potential of the GBD is positive, due to the presence of cationic amino acids (Figure 2B). Once the association is achieved on the basis of electrostatic forces, the gangliosides of the raft and the amino acid side chains of the GBD modify their conformation, allowing the establishment of hydrogen bonds [14]. This mutual adaptation stabilizes the virus on the raft and triggers the unmasking of the RBD. This process can be compared to a domino effect [77,78] that propagates through the trimer like a conformational wave [79,80]. This is reminiscent of the interfacial activation of many lipases at the lipid–water interface [60,81]. Examples of amino acid movements controlled by gangliosides are shown in Figure 2C.

## 3. Evolution of the SARS-CoV-2 GBD over Six Years (2019–2025)

**Figure 3:**
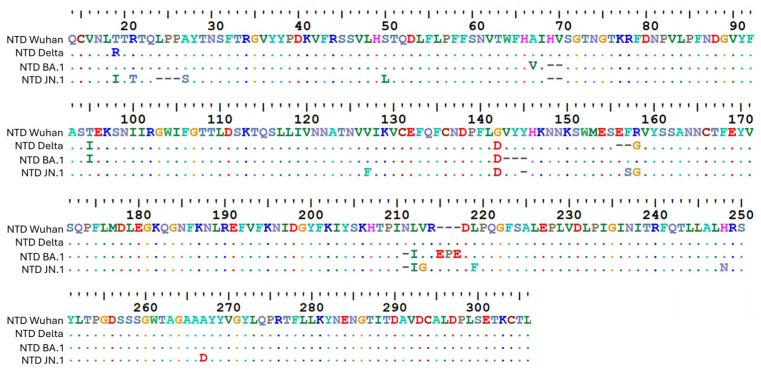
Amino acid sequence alignment of the NTD of Delta, BA.1, and JN.1 variants with the Wuhan strain used as a reference. Mutations in the NTD of the Delta variant (B.1.617.2): T19R T95I G142D ∆E156 ∆F157 R158G [89]. Mutations in the NTD of Omicron BA.1 (first genetic leap): A67V ∆H69 ∆V70 T95I G142D ∆V143 ∆Y144 ∆Y145 ∆N211 L212I Ins214EPE [89]. Mutations in the NTD of Omicron JN.1 (second genetic leap, started with BA.2.86): T19I R21T A27S ∆L24 ∆P25 ∆P26 S50L ∆H69 ∆V70 V127F G142D ∆Y144 ∆N211 F157S R158G L212I V213G L216F H245N A264D [90]. Amino acid alignments were performed with Mafft (version 7) (https://mafft.cbrc.jp/alignment/server/index.html, accessed on 13 November 2025) and visualized with BioEdit (version 7.7.1.0) (https://thalljiscience.github.io/, accessed on 13 November 2025).

When it appeared in Europe in the first months of 2020, the virus acquired exacerbated mutational properties due to a point mutation (P323L) in the gene coding for the RNA polymerase [66,83,84]. This mutation gave more freedom to the enzyme–substrate complex and therefore reduced fidelity, which facilitated the appearance of new variants in a pandemic context [66,83,85]. The localization of the NTD at the interface of the viral envelope and the host cell plasma membrane promotes the accumulation of mutations in successive variants of SARS-CoV-2 [86,87,88]. To illustrate this principle, we can first compare the amino acid sequences of the NTDs of these variants. An alignment of the amino acid sequence of the NTD of Delta, Omicron BA.1, and Omicron JN.1 variants with the Wuhan reference strain is shown in Figure 3.

## 4. The Q134-F135-N137 Triad Is Fully Conserved After 6 Years of SARS-CoV-2 Evolution

**Figure 4:**
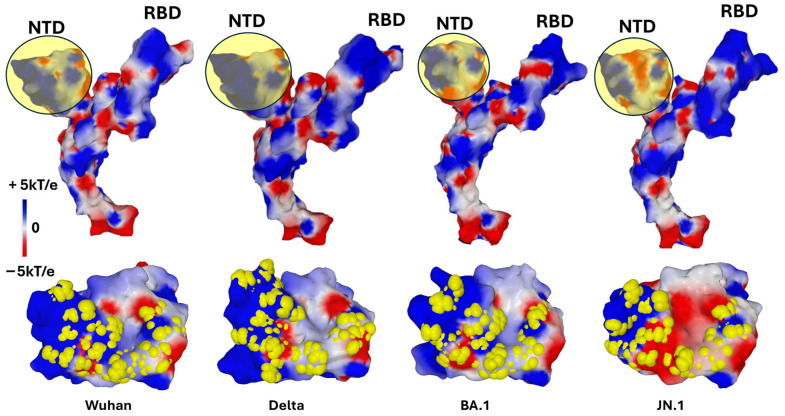
Evolution of the SARS-CoV-2 Spike protein over six years (2019–2025). Structural changes in the Spike protein can be visualized by the surface electrostatic potential. In the top panel, a yellow-colored disk has been superimposed on each NTD to better visualize the changes in electrostatic potential. The NTD of Delta has a more electropositive potential than that of Wuhan, while those of the Omicron lineage (BA.1 and JN.1) have reversed to a more electronegative surface. These variations are offset by the electrostatic potential of the RBD which has been steadily increasing since the emergence of SARS-CoV-2 in 2019. In the bottom panel, the amino acids constituting the GBD of each variant are represented in atomic yellow spheres, illustrating the remarkable adaptation of the raft-binding capacity of the NTD. Protein structures and surface potential were generated with MMV.

How does the virus accumulate these mutations while remaining infectious? This phenomenon demonstrates continuous adaptation, likely orchestrated by a complex dialog (“cross-talk”) with the host cell plasma membrane, notably through lipid raft microdomains [14]. This selection process took into account the two critical parameters for the Spike–raft interaction: the electrostatic potential of the NTD [91] and the capacity of the mutated GBD to adapt its conformation to the molecular quicksand that is characteristic of the raft [14]. Thus, we observed a progressive increase in the surface potential of the NTD from the original Wuhan strain to the Delta variant, which seems to have reached the maximum possible level (Figure 4). From the Omicron lineage, another logic prevailed, with a transfer of the electropositive potential from the NTD to the RBD. The price to pay for this evolutionary leap was significant, with a rounding of the NTD surface for the first Omicron variant (BA.1) [89] which was corrected from the following variant (BA.2) [92] after an intermediate Delta–Omicron recombination attempt (Deltamicron) [93]. A second evolutionary leap led to the Omicron variants circulating today, including JN.1 [94,95], a strain derived from BA.2.86 [90]. All these variants retained the ability of the original strain to interact with raft gangliosides, without any loss of functionality (Figure 4).

In a manner unrelated to these adaptive changes, an intriguing triad of amino acids—Q134, F135, and N137—was previously identified as an invariant spot on the NTD surface during the first two years of SARS-CoV-2 evolution [96,97]. Molecular dynamics simulations performed independently by two laboratories, ranging from all-atom 50 ns [55] to coarse grain 10 µs protocols [33,54], remarkably converged to identify this triad as a major component of the GBD on the NTD surface. To provide an up-to-date estimation, we analyzed the presence of the triad Q134, F135, and N137 across all SARS-CoV-2 spike sequences available in the COVID-19 Viral Genome Analysis Pipeline of the Los Alamos database [https://cov.lanl.gov/content/index, accessed on 12 November 2025]. This query demonstrated a remarkable conservation of each of these amino acids. Among over 7 million sequences analyzed, only a few hundred divergences were identified. Therefore, we can conclude that the triad, previously identified as an invariant spot on the NTD surface since 2020, has remained conserved across successive SARS-CoV-2 variants, including the currently circulating JN.1 variant [98] (Figure 3).

**Figure 5:**
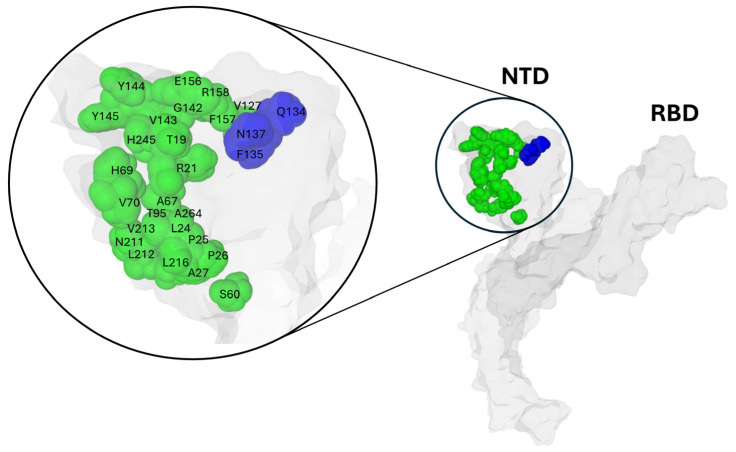
Structural Mapping of the SARS-CoV-2 Wuhan Spike Protein: Highlighting the Conserved Q134-F135-N137 Triad (colored blue) and Variant Mutation Sites (colored green). Protein structures were generated with MMV.

On the NTD surface, the Q134-F135-N137 triad is spatially close to numerous mutation sites detected on successive variants (Figure 5).

In contrast, the triad clearly defines an invariant spot on the NTD surface, marking a boundary between a region of high variability and a more conserved region. What is surprising is that both regions, the variable and the conserved, are involved in the interaction with the rafts [96]. For instance, the conserved Q134-F135-N137 triad is very close spatially to a mutation-prone spot, E156-F157-R158. The juxtaposition of a highly mutable region and a small invariant core emphasizes the enigmatic nature of the Q134-F135-N137 triad.

**Figure 6:**
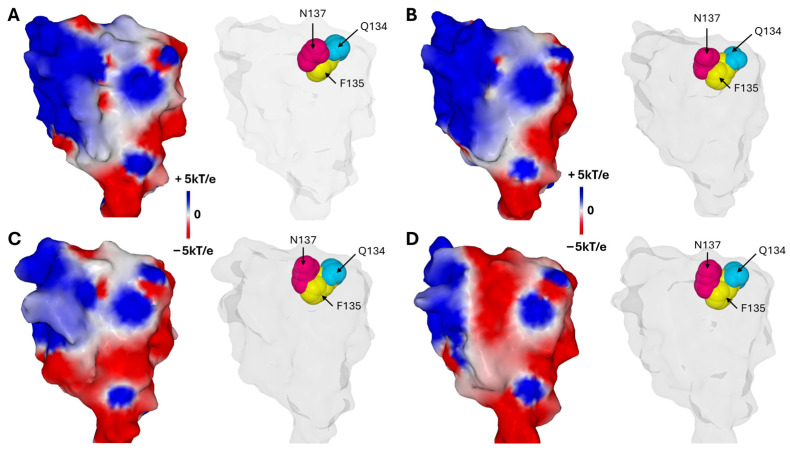
The Conserved Q134-F135-N137 Triad and Its Surrounding Electrostatic Landscape Variability Among SARS-CoV-2 Variants. (**A**)—Wuhan strain. (**B**)—Delta variant. (**C**)—Omicron BA.1 variant. (**D**)—Omicron JN.1 variant. In each case the left panel illustrates the electrostatic surface potential of the NTD and the right panel shows the Q134-F135-N137 Triad. Note that the aromatic ring of F135 points towards the inside of the NTD while the side chains of Q134 and N137 point in the opposite direction, towards the raft. Protein structures and surface potential were generated with MMV.

The amino acid composition of this triad combines polar and apolar residues. In the unbound conformation, the aromatic ring of the central residue, F135, projects toward the interior of the NTD, whereas Q134 and N137 flank F135 with their polar side chains oriented outward toward the raft (Figure 6). This unique configuration, together with its conservation after six years of virus evolution, supports the hypothesis that the triad plays a critical and possibly irreplaceable role in spike function, justifying focused structural and functional studies to unravel its role [97].

## 5. Electrostatic Landscape Variability Among SARS-CoV-2 Variants

An intriguing observation is that the surface electrostatic potential of the N-terminal domain (NTD) significantly evolves across successive SARS-CoV-2 variants, while the Q134-F135-N137 triad remains strikingly conserved (Figure 6). It is important to note that the surface potential maintains a minimum level of electropositive zones necessary for interaction with the host cell’s lipid rafts. During the Wuhan to Delta evolution, the NTD surface potential became more electropositive. This trend then reversed starting at Omicron BA.1, with other regions of the trimeric spike compensating for this evolutionary shift [13]. This observation reinforces the contrast between the invariability of the triad and the other regions of the NTD subject to substantial mutational pressure. Such a pattern highlights the functional importance of the triad, which resists mutational fluctuations of adjacent regions on the NTD surface, presumably to preserve critical interactions with lipid raft gangliosides essential for viral attachment.

## 6. A Conformational Wave Within the NTD

**Figure 7:**
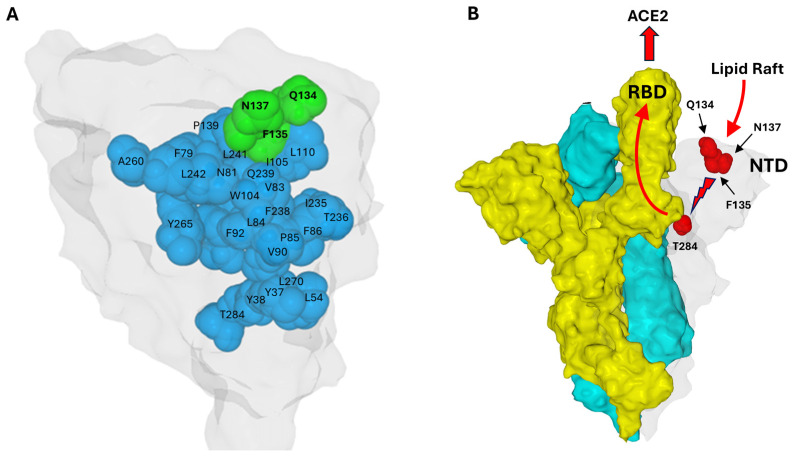
Structural Continuum of the Conformational Wave Initiated by the Q134-F135-N137 Triad Extending to Position 284 in the Wuhan Spike Protein. (**A**). The NTD surface is colored light gray. The Q134-F135-N137 triad is colored green, the amino acids involved in the conformational wave are blue. (**B**). Proposed mechanism: A conformational wave initiated by the NTD–lipid raft interaction (light gray protomer) propagates from residue F135 to T284. In the trimeric structure, T284 makes direct contact with the adjacent protomer (colored yellow), triggering the unmasking of its RBD to enable ACE2 binding. The third protomer is colored cyan. In this respect, T284 acts as a conformational transducer that connects the NTD of one protomer and the RBD of the neighbor protomer. Residues Q134, F135, and N137 are solvent-exposed (surface-accessible), whereas the other residues discussed are buried in the protein interior (core/internal residues). Protein structures were generated with MMV.

To understand why the Q134-F135-N137 triad is conserved, the amino acids in direct contact with these residues were analyzed, using the Swiss-Pdb Viewer software version 4.1.0 with a step-by-step method. The first amino acid considered was F135. All amino acids in contact with the aromatic ring of this phenylalanine were identified. A similar analysis was then performed on these amino acids, and so on, with a cutoff distance < 5 Å. By identifying amino acids in direct contact with F135, and proceeding stepwise from one contact to the next, a structural continuum emerges connecting the triad at the surface to residue T284, located over 40 Å away (Figure 7A). The shortest route identified involved Q134-F135-I105-Q239-W104-F92-V90-L270-L54-Y37-Y38-T284. This observation supports a domino-like mechanism, or conformational wave, initiated at the triad and propagating through the NTD interior to reach regions contacting the receptor-binding domain (RBD) of an adjacent protomer (Figure 7B). This structural continuum suggests a pathway by which the conserved triad could regulate longer-range conformational changes essential for spike activation and receptor engagement. This mechanistic hypothesis aligns with emerging insights on allosteric regulation in viral glycoproteins through networks of intramolecular interactions. In this model, T284 acts as the final transducer, bridging the NTD of one protomer to residue L560 in the receptor-binding domain (RBD) of the adjacent protomer through specific van der Waals interactions (Figure 7B). This mechanical coupling is highlighted by structural changes between states: in the closed prefusion conformation, T284 and L560 are separated by 3.56 Å; however, in the open conformation characterized by an unmasked RBD, these residues move significantly closer to a distance of only 2.25 Å.

**Figure 8:**
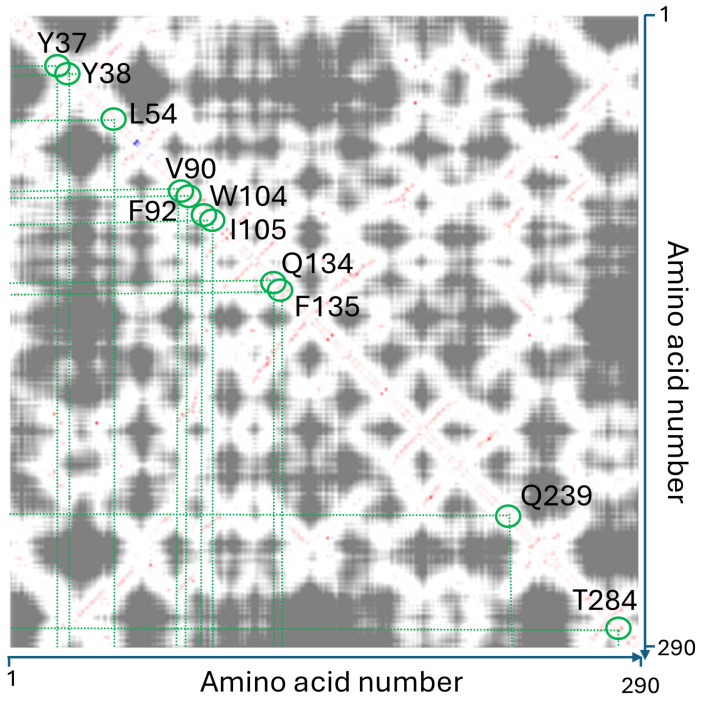
Interaction Energy Matrix (IEM) analysis. The pairwise interaction energy matrix (derived from IEM web server: https://mu-pub-245-249.flt.openstack.cloud.e-infra.cz/energy/, accessed on 10 December 2025) identifies thermodynamically significant couplings between residues. The diagonal represents local sequence contacts, while the off-diagonal points (highlighted in red) reveal long-range allosteric couplings. The red path traces the conformational wave, confirming that the identified wave is energetically coupled across the domain. This combined matrix provides an interaction energy matrix enhanced by the distance (proximity) measure. The attractive interactions are shown in red, the repulsive in blue and their strength is indicated by saturation of the color. Additionally, the distance of the residues is coded in the shades of gray. The close residues are plotted in light (e.g., T284 and Y38) and the shade darkens with increasing distance.

The structural integrity of the conformational wave was primarily defined through rigorous manual inspection of inter-residue distances, a procedure that was implemented to preclude software-dependent artifacts. These manual determinations were subsequently corroborated by automated analysis using RING [99] and the Interaction Energy Matrix (IEM) [100,101]. Notably, the IEM analysis demonstrated that these residues are not merely packed together by chance but share favorable interaction energies, creating a stable thermodynamic path for signal propagation (Figure 8).

**Figure 9:**
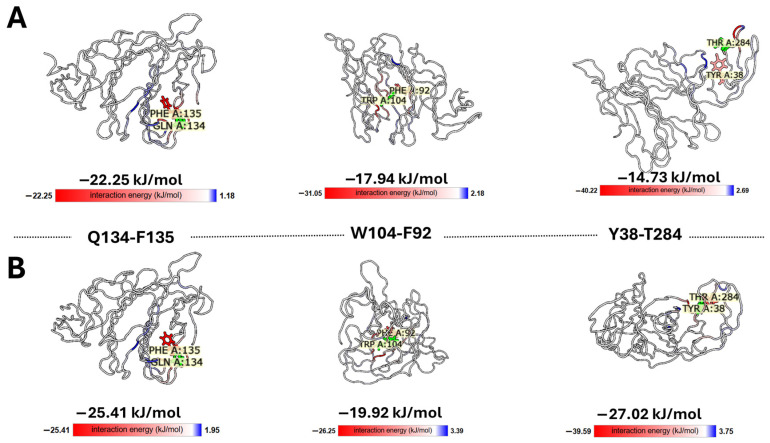
Energetic decomposition of the conformational wave pathway. Representative snapshots show the pairwise interactions identified by the distance-based conformational wave analysis. Interaction energies (in kJ/mol) for each pair were calculated using the Interaction Energy Matrix (IEM) server. The cartoons were extracted from the 3D structure viewer of IEM. The pairwise interaction energy mode assigns the colors in relation to the reference residue, and the saturation of the color indicates the strength of the individual pairwise interactions between the reference (in green) and other residues. The stabilizing interactions are shown red, repulsion in blue. In this mode, the most stabilizing residues can be identified on the model visually, because the colors are saturated according to the strength of the interactions. (**A**). closed, unbound conformation. (**B**). open conformation after ganglioside binding.

To quantify the thermodynamic stability of the proposed pathway, we extracted pairwise interaction energies from the IEM (Figure 9). The analysis reveals that the wave path is supported by a series of energetically favorable interactions ranging. Notably, the Q134-F135 pair exhibits a strong interaction energy of −22.25 kJ/mol, validating its role as a stable hub. The variation in energy magnitudes—alternating between strong ‘anchors’ (e.g., T284-Y38) and more flexible contacts (e.g., L54-L270)—is consistent with the requirements for a pathway that must be both stable enough to exist and flexible enough to transmit conformational signals. Finally, the effectiveness of the conformational wave is demonstrated by the redistribution of interaction energies. While the cumulative interaction energy of the wave network remains nearly isoenergetic between states (~−150 kJ/mol), the local distribution shifts dramatically. Specifically, the terminal interaction pair Y38-T284 strengthens significantly from −14.73 kJ/mol (unbound) to −27.02 kJ/mol (bound).

This indicates that the conformational wave functions not by destabilizing the domain, but by redistributing enthalpy—channeling stability to the effector site to ‘lock’ the bound conformation. This precise energetic focusing, within a globally stable network, confirms that the identified residues constitute a bona fide, thermodynamically efficient allosteric pathway. In other words, the wave acts as an isoenergetic energy transfer system, doubling the energy of interaction of the final pair (Y38-T284) without a global energy cost.

**Figure 10:**
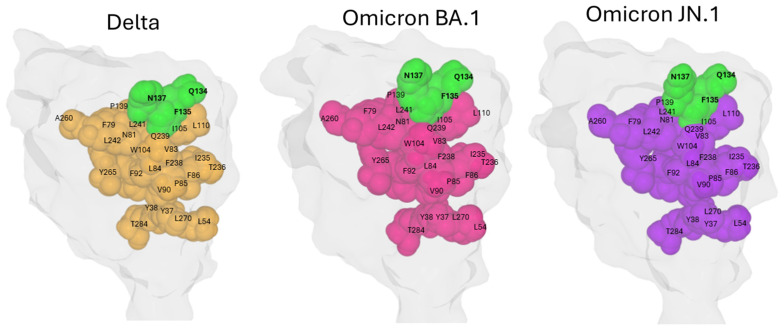
Comparative Mapping of the Conformational Wave Network from the Conserved Triad to Position 284 in SARS-CoV-2 Spike Variants Delta, Omicron BA.1, and Omicron JN.1. Residues Q134, F135, and N137 are solvent-exposed (surface-accessible), whereas the other residues discussed are buried in the protein interior (core/internal residues). Protein structures were generated with MMV.

The presence of the structural continuum linking the conserved triad Q134-F135-N137 and residue T284 was then assessed through analysis of the NTD structures of the Delta, Omicron BA.1, and Omicron JN.1 variants. These three variants span six years of SARS-CoV-2 evolution. In all cases, the amino acids implicated in this conformational wave are well conserved and, most importantly, structurally maintained (Figure 10). This observation highlights the robust preservation of the allosteric network despite extensive mutational changes accumulated in the NTD over time. Hence, the conformational wave from the Q134-F135-N137 triad to deeper NTD regions appears to be a fundamental structural feature important for spike function across SARS-CoV-2 variants. This analysis reinforces the concept of the Q134-F135-N137 triad as a conserved anchor within a dynamic mutational landscape, linking surface interactions with internal conformational dynamics essential for viral infectivity.

The distance between coupled amino acids is less than 5 Å in all four viruses analyzed (including Wuhan taken as reference), providing a structural basis for a domino-like coupling mechanism propagating from the NTD surface to the deeper regions of the domain. This spatial arrangement supports the hypothesis of a conformational wave mediating long-range allosteric communication essential for spike protein function and viral infectivity. This is in line with structural bioinformatics findings where direct residue contacts facilitating allosteric networks are defined by such Å-scale distances [102,103,104,105].

**Figure 11:**
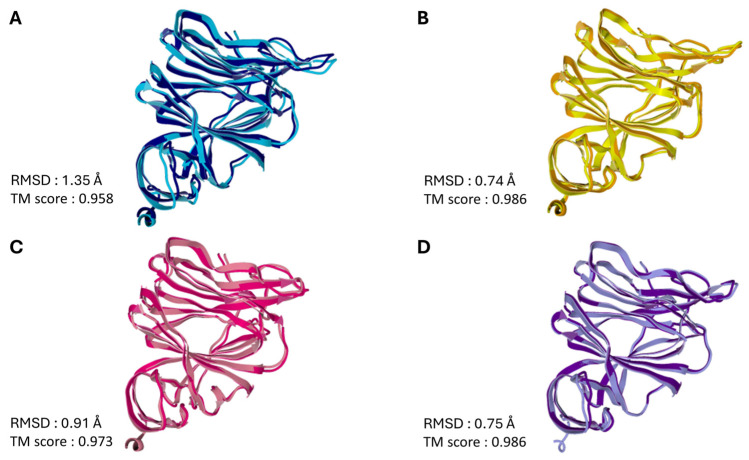
Insight into NTD Structural Dynamics: Superimposition of SARS-CoV-2 Spike Domain with and without GM1 Lipid Raft Interaction. (**A**)—Wuhan; (**B**)—Delta; (**C**)—BA.1; (**D**)—JN.1. In each case, the secondary structure of the NTD before interaction with the GM1 ganglioside raft is rep-resented in a lighter color, after interaction with the raft in a darker color. The PDB files were uploaded in the ZhangLab web page (https://zhanggroup.org/TM-score/, accessed on 23 October 2025) which calculates the RMSD and TM score values for two related proteins. The TM-score is a quantitative metric used to assess the topological similarity between protein structures. The score ranges from 0 to 1, where a value of 1 indicates a perfect structural match. According to statistical analyses of protein structures in the Protein Data Bank (PDB), TM-scores below 0.17 correspond to pairs of unrelated proteins, while scores above 0.5 generally indicate that the structures share the same fold classification [113].

To visualize the propagation of conformational changes controlled by the conserved triad, we superimposed the NTD structures before and after interaction with the ganglioside GM1 lipid raft. This superimposition is presented for the four SARS-CoV-2 viruses and variants analyzed in this review, encompassing the evolutionary period from 2019 to 2025 (Figure 11). The superposition of the bound and unbound NTD structures yields high TM scores (0.958–0.986), indicating that the global topology of the domain is preserved upon GM1 interaction [106]. This preservation of the overall fold suggests that the observed conformational wave does not involve large-scale unfolding or secondary structure remodeling [107,108]. Instead, the mechanism relies on subtle, coordinated displacements of the backbone and side chains—akin to elastic deformation [109,110]—that are sufficient to transmit allosteric signals across the domain without compromising structural stability [111,112].

**Figure 12:**
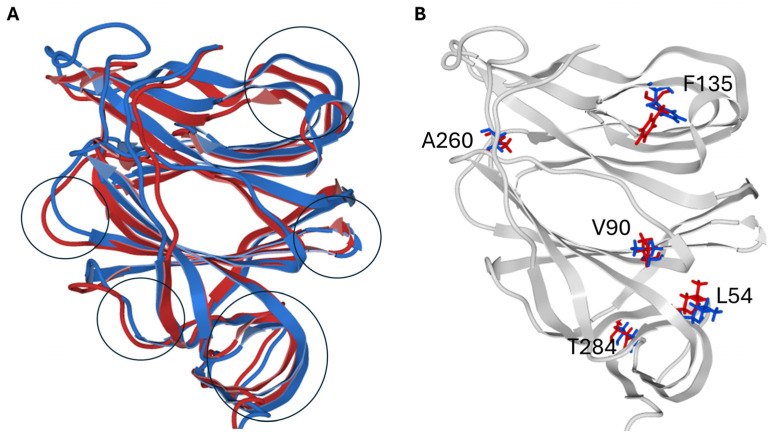
Structural Superposition of SARS-CoV-2 Spike NTD in Close and Open States. (**A**). Superposition of the secondary structure of the NTD in the close conformation (colored blue) and in the open conformation (colored red). The main conformational rearrangements are marked with black circles. (**B**). Structural rearrangements observed in key amino acid residues of the conformational wave (same color code as in panel (**A**)). Note that the most critical change concerns the aromatic cycle of F135 which tilts towards the interior of the NTD and triggers the conformational wave. For clarity, only the secondary structure of the close conformer is shown (colored gray). For clarity, only critical residues involved in the conformational wave are shown.

The structural superimposition of the NTD of the spike protein in its metastable closed (RBD still masked, pdb 6VSB) and open (RBD unmasked, pdb 7DK3) conformations is shown in Figure 12, with spike structures obtained by cryo-electron microscopy. This comparison visualizes how the central residue of the conserved triad (F135) and the conformational wave link to large-scale structural shifts, validating the changes induced by lipid raft interaction. These rearrangements underscore the coordinated interplay between subunits essential for membrane fusion and viral infectivity. The data align well with cryo-electron microscopy structures that describe the stepwise structural transition from pre- to post-fusion states crucial for viral fusion machinery activation [37].

## 7. Hypothesis: A Quantum Wave Within the NTD?

Aromatic residues play a key role in the attachment and/or entry mechanisms of several viruses, including coronaviruses [114,115], retroviruses [116,117] and influenza virus [118]. Interestingly, the aromatic rings of phenylalanine residues can undergo 180° flips, which may contribute to conformational fluctuations that influence these early steps of viral entry [119]. Indeed, phenylalanine residues have been implicated in conformational waves associated with the functional activation of proteins [120,121]. A phenylalanine residue (F113) in lipases acts as a dynamic switch, flipping to enable interfacial activation and substrate access via aromatic rearrangements [81]. This mirrors our finding that F135 drives trimer toggling between closed/open states in SARS-CoV-2 Spike trimers. Conserved across lipid-interacting proteins, these Phe mechanisms highlight shared evolutionary strategies targetable by small molecules disrupting hydrophobic packing. But there is more: aromatic cycles can facilitate intramolecular electron transfer, as observed in protein electron transport chains. This quantum mechanism has been proposed to explain how magnetic-sensory molecules such as cryptochromes allow light-mediated magnetoreception in the retina of migrating birds [122,123,124]. In cryptochromes, light activates flavin adenine dinucleotide (FAD), triggering sequential electron transfer primarily along a tetrad of tryptophan residues to form radical pairs sensitive to Earth’s magnetic field. This radical pair mechanism enables magnetoreception in migratory birds’ retinas, where spin dynamics in FAD^•−^-TrpH^•+^ radical pairs respond to magnetic inclination [125]. 

**Figure 13:**
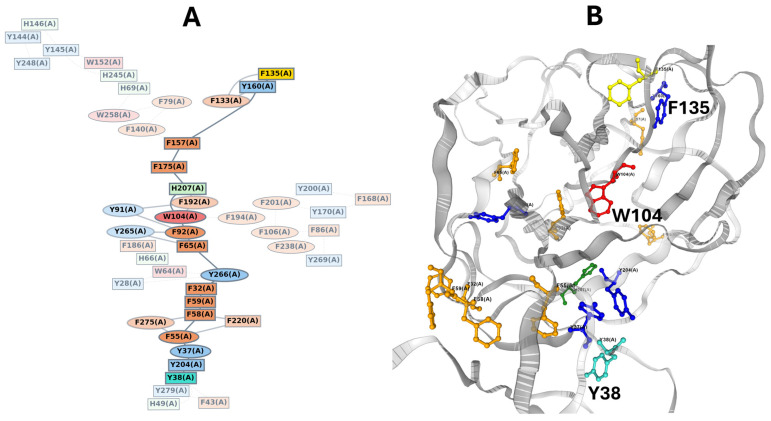
Identification of a putative quantum/electron transfer pathway superposed on the conformational wave. (**A**). Graph theory representation of the electron transfer pathway. The graph, generated by the eMAP server, identifies a continuous network of electron-transfer-active moieties. (**B**). Structural mapping of the quantum wave. The identified ETA pathway is mapped onto the 3D structure. The pathway (colored sticks) proceeds from F135 through a hydrophobic aromatic bridge involving W104 (red), effectively wiring the conformational wave to an electronic tunneling route. The high density of aromatic residues (Trp, Tyr, Phe) along the conformational path suggests a dual mechanism where allosteric signal propagation may be coupled to charge transfer or electronic delocalization events linking F135 and Y38. Data were obtained via https://emap.bu.edu/ (accessed on 10 December 2025).

In the case of the SARS-CoV-2 spike protein, the available cryo-electron microscopy structures reveal numerous aromatic residues, raising the intriguing possibility of an electron transfer chain propagating through the aromatic residues of the NTD. Indeed, proteins without metal cofactors can support long-range electron transport via hopping between aromatic residues [126,127]. To investigate this hypothesis, we used the eMap server, a web application for identifying and visualizing electron or hole hopping pathways in proteins [128]. The program identified a continuous chain connecting F135 to Y38, with the pathway passing through the central hub W104 and terminating near the conformational transducer residue T284 (Figure 13). This superposition of a quantum wave (electronic pathway) onto the conformational wave (mechanical pathway) suggests that the allosteric mechanism may involve coupled electron-proton transfer or rapid electronic signaling that precedes the slower conformational shift. The spatial arrangement of aromatic residues in the NTD is likely not random but constitutes a viable “molecular wire” capable of sustaining long-range electron or hole transfer via quantum tunneling. This hypothesis warrants further investigation through experimental validation, such as site-directed mutagenesis of key aromatic residues [129,130] or time-resolved spectroscopy to detect transient charge-transfer states [131,132].

## 8. Biological Significance

This review, based on a broad range of published in silico and experimental data, elucidates the pivotal role of gangliosides, integral components of lipid rafts, in facilitating the early stages of SARS-CoV-2 infection. These negatively charged glycosphingolipids serve as electrostatic attractors for the virus, mediating the initial docking of the spike protein via a large flat surface of the NTD. The NTD is divided into two parts, one with strong mutational capacity and one much more conserved. In the preserved portion, the Q134-F135-N137 triad attracted our attention due to its intriguing composition mixing polar amino acids (Q134 and N137) and an aromatic amino acid (F135) [97]. Its remarkable conservation within a context of widespread mutations in vicinal regions of the NTD suggests a critical functional role extending beyond basic viral attachment. We propose a novel paradigm whereby the triad not only anchors the virus to host gangliosides in lipid rafts but also initiates a conformational wave traversing the NTD interior. This allosteric wave is hypothesized to orchestrate the exposure of the receptor-binding domain (RBD) on the neighboring protomer—an indispensable conformational transition underpinning efficient engagement with the angiotensin-converting enzyme 2 (ACE2) receptor and subsequent viral entry. The maintenance of such a sophisticated molecular mechanism likely provides SARS-CoV-2 with a selective advantage: it balances antigenic flexibility to evade host immune responses while preserving critical structural features essential for infectivity. This duality underscores the spike protein’s evolutionary ingenuity in navigating host defenses and optimizing transmissibility.

It is interesting to note that transmembrane proteins like plasmolipin (PLLP) also interact with gangliosides via sialic acid negativity, ordering disordered regions and propagating conformational waves across the membrane [79]. Quantum waves underpin this through π-π stacking and delocalized electrons in aromatic motifs, enhancing stability and electron transfer efficiency. This dual-wave model exemplifies epigenetic modulation [133], where lipid rafts translate quantum fluctuations into functional conformational changes, with implications for myelin stability and viral protein dynamics like SARS-CoV-2 spike. Ultimately, the integration of these physical constraints with quantum mechanisms—as recently proposed by Fantini et al. within the framework of the “fundamental parameters of biology” [134], further challenges gene-centric views, revealing membrane-associated proteins as quantum-entangled lipid–protein assemblies. 

## 9. Conclusions

By analyzing the different results obtained in silico and experimentally, we provide new mechanistic explanations inherent to the modes of interaction of SARS-CoV-2 with raft gangliosides. First, despite the mutational variability of the NTD interface in successive variants of the virus, two mechanisms ensure the capacity to bind to the rafts: (i) An adaptation of the surface electrostatic potential combined with the establishment of hydrogen bonds between the NTD and the gangliosides: the amino acids may change, but the interaction is still possible. (ii) The existence on the NTD surface of a zone excluding mutations, the boundary of which is constituted by the conserved triad Q134/F135/N137. (iii) By identifying and characterizing this enigmatic triad within the NTD of the spike protein, we uncover an invariant molecular hotspot critical for virus–host interaction despite extensive genetic variability. (iv) Structural data reveals a structural continuum of amino acid residues linking this triad and the point of contact with the RBD of a neighboring protomer. (v) A conformational wave initiated at the triad by contact with the raft propagates through the NTD and transmits conformational information to the neighboring protomer. (vi) This mechanism exemplifies how SARS-CoV-2 balances evolutionary adaptation with preservation of essential functional elements. Overall, the triad represents promising targets for antiviral intervention. Disrupting this conserved structural pathway could impair spike activation, thwart viral entry, and provide a strategy resilient to emerging variants. For instance, peptidomimetics or macrocycles designed to mimic the Q134-F135-N137 motif would competitively bind to the triad’s interacting partners, effectively silencing the conformational transducer at its source.

## 10. Future Perspectives

While the conservation of the Q134-F135-N137 triad across SARS-CoV-2 variants strongly suggests functional importance, we acknowledge that conservation alone does not constitute proof of indispensability. Direct loss-of-function experiments [114], such as site-directed mutagenesis of individual residues (Q134A, F135A, N137A) or combined mutations, would be necessary to definitively establish the functional contribution of each residue to ganglioside binding and viral infectivity.

Future research directions should focus on high-resolution structural characterization of spike-ganglioside complexes across emerging variants to elucidate the fine molecular details governing this conserved mechanism. Additionally, in vitro and in vivo validation of inhibitors targeting the triad or the conformational/quantum wave will be critical to assess feasibility and efficacy. Exploration of lipid raft modifiers or ganglioside mimetics as therapeutic agents could complement these approaches. Further understanding of how host membrane composition modulates spike dynamics during infection may open broader perspectives in viral pathogenesis and host adaptation.

## Data Availability

No new data were created or analyzed in this study.

## Abbreviations

The following abbreviations are used in this manuscript:

**ACE-2**	Angiotensin-Converting Enzyme 2
**FAD**	Flavin adenine dinucleotide
**GBD**	Ganglioside-binding domain
**MMV**	Molegro Molecular Viewer
**NADPH**	Nicotinamide adenine dinucleotide phosphate
**NTD**	N-terminal Domain
**RBD**	Receptor-binding Domain
**SARS-CoV-2**	Severe Acute Respiratory Syndrome Coronavirus 2
